# Polyhexamethylene Biguanide and Nadifloxacin Self-Assembled Nanoparticles: Antimicrobial Effects against Intracellular Methicillin-Resistant *Staphylococcus aureus*

**DOI:** 10.3390/polym10050521

**Published:** 2018-05-12

**Authors:** Nor Fadhilah Kamaruzzaman, Maria de Fatima Pina, Alexandru Chivu, Liam Good

**Affiliations:** 1 Department of Pathobiology and Population Science, Royal Veterinary College, University of London, Royal College Street, London NW1 0TU, UK; lgood@rvc.ac.uk; 2 Present address: Faculty of Veterinary Medicine, Universiti Malaysia Kelantan, Locked Bag 36, Pengkalan Chepa, 16100 Kota Bharu, Kelantan, Malaysia; 3 University College London School of Pharmacy, 29-39 Brunswick Square, Bloomsbury, London WC1N 1AX, UK; mfatimagpina@gmail.com; 4 UCL Centre for Nanotechnology and Regenerative Medicine, Division of Surgery & Interventional Science, University College London, London NW3 2PF, UK; a.chivu.14@ucl.ac.uk

**Keywords:** skin and soft tissue infections, polyhexamethylene biguanide, nadifloxacin, nanoparticles, intracellular MRSA

## Abstract

The treatment of skin and soft tissue infections caused by methicillin-resistant *Staphylococcus aureus* (MRSA) remains a challenge, partly due to localization of the bacteria inside the host’s cells, where antimicrobial penetration and efficacy is limited. We formulated the cationic polymer polyhexamethylene biguanide (PHMB) with the topical antibiotic nadifloxacin and tested the activities against intracellular MRSA in infected keratinocytes. The PHMB/nadifloxacin nanoparticles displayed a size of 291.3 ± 89.6 nm, polydispersity index of 0.35 ± 0.04, zeta potential of +20.2 ± 4.8 mV, and drug encapsulation efficiency of 58.25 ± 3.4%. The nanoparticles killed intracellular MRSA, and relative to free polymer or drugs used separately or together, the nanoparticles displayed reduced toxicity and improved host cell recovery. Together, these findings show that PHMB/nadifloxacin nanoparticles are effective against intracellular bacteria and could be further developed for the treatment of skin and soft tissue infections.

## 1. Introduction

Skin and soft-tissue infections (SSTIs) affect a wide range of patients including elderly and hospitalized individuals with immunocompromised conditions, and individuals with skin injuries who have frequent skin to skin contact, such as athletes [1,2]. Clinical manifestation of SSTIs can range from mild to life-threatening infections, with more than 70% of cases requiring hospitalization [3].

*Staphylococcus aureus* (*S. aureus*) is the leading pathogen causing SSTIs worldwide [4,5,6]. *Staphylococcus aureus* is a Gram-positive pathogen that colonizes between 15 to 40% of healthy individuals in their nose, skin, and mucous membranes [7]. Infections by *S. aureus* were once easy to treat by the administration of beta-lactam antibiotics. However, the rise of methicillin-resistant *S. aureus* (MRSA)-associated infections worldwide has led to resistance against available antibiotic therapy, increasing morbidity and mortality in hospitalized patients [8,9]. Additionally, *S. aureus* is known to invade and survive in the host’s cell, and in this state, they are further protected from the immune surveillance and antimicrobial therapy. These protection mechanisms can lead to persistent infections, which may increase transmission and mortality [10]. Many antimicrobials do not effectively treat intracellular infections due to (1) poor penetration and retention of the host cells; (2) reduced potency in intracellular acidic environments and localization in the different cell compartments where bacteria reside; and (3) reduced potency towards slow-growing intracellular bacteria [11,12,13,14,15,16]. These factors are believed to contribute to therapeutic failure.

The development of a new antimicrobial is a lengthy and expensive process. An alternative approach is to improve the overall potency of available antimicrobials through formulation. Advances in nanotechnology offer new possibilities to improve potency and reduce toxicity. Nanoparticles can be prepared using synthetic or natural substances and their features include large surface areas and functional chemical groups that can be utilized in combination with active pharmaceutical ingredients (APIs) [17]. Physical encapsulation, adsorption or chemical conjugation of drugs into nanoparticles can significantly improve the therapeutic index [18]. For example, Miramoth et al. [19] demonstrated that squalenoylated penicillin nanoparticles are more effective against intracellular *S. aureus* in macrophages in comparison to free penicillin. Clemens et al. [20] showed that rifampicin loaded into mesoporous silica-polyethyleneimine nanoparticles are more potent towards intracellular *Mycobacteria tuberculosis*, in comparison to the free drug. Such examples demonstrate that combinations of antimicrobials in nanoparticulate formulations can potentially improve antimicrobial potency. Additional advantages of the application of nanoparticles for drug loading and delivery have been described in References [21,22,23,24,25,26,27].

**Figure 1:**
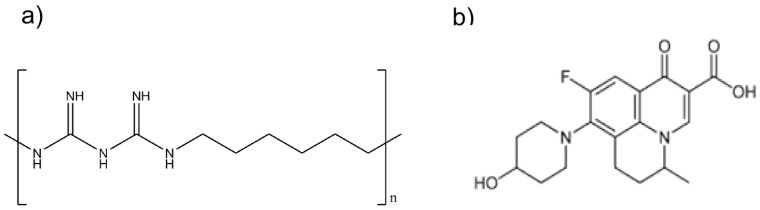
Structure of (**a**) that polyhexamethylene biguanide (PHMB) and (**b**) nadifloxacin.

We recently reported that polyhexamethylene biguanide (PHMB)—a cationic antimicrobial polymer (see Figure 1a)—can bind to nucleic acids, forming nanoparticles in vitro [28]. Additionally, we demonstrated that PHMB can form nanoparticles and improve delivery of CpG oligonucleotide (ODN), an immunomodulatory, into macrophages [29,30]. We also demonstrated that the polymer can enter a range of mammalian cells and kill intracellular bacteria and parasites [31]. Therefore, PHMB characteristics provide an exciting platform for formulation with antimicrobials, potentially improving antimicrobial cell delivery and controlled release, as well as providing direct polymer-mediated antimicrobial effects. We also showed that nadifloxacin (Figure 1b)—a topical antimicrobial that is currently administered for the treatment of skin infections—has antimicrobial activities against intracellular *S. aureus* [31]. With this knowledge, we aimed to further exploit PHMB by examining its possible interaction with nadifloxacin to form nanoparticles, and to further determine the antimicrobial activities of the nanoparticles against intracellular *S. aureus* in infected keratinocytes and to characterize the toxicity profile against the host cells.

Polyhexamethylene biguanide is a cationic polymer of repeating hexamethylene biguanide groups, with *n* average = 10–12 (*n* is the number of structural unit repeats) and a molecular weight of (*MW*) 3025 g/mol. Nadifloxacin is a topical fluoroquinolone with *MW* 360 g/mol.

## 2. Materials and Methods

### 2.1. Preparation and Optimization of PHMB/Nadifloxacin Nanoparticles

Polyhexamethylene biguanide was obtained from Tecrea Ltd., London, UK and nadifloxacin was obtained from Santa Cruz Biotechnology, UK. All antimicrobials were prepared in stock solution at 10 mg/mL. PHMB was dissolved in sterile distilled water, and nadifloxacin was dissolved in 0.1 M sodium hydroxide solution.

The PHMB/nadifloxacin nanoparticles were prepared by the self-assembly method [28]. In brief, 0.5 mL of PHMB (80 mg/L in water) and 0.5 mL of nadifloxacin (160 mg/L in 0.1 M NaOH) were mixed in a 1.5 mL microcentrifuge tube followed by incubation in an incubator shaker at 200 rpm for 15 min.

### 2.2. Physical Characterization of PHMB/Nadifloxacin Nanoparticles

#### 2.2.1. Size and Zeta Potential Measurements

Intensity mean hydrodynamic size and zeta potential of PHMB and PHMB/nadifloxacin nanoparticles were measured on a Malvern Zetasizer–NanoZS (Malvern Instruments, Malvern, UK) with a He–Ne laser (wavelength of 632.8 nm). The measurements were carried out at a scattering angle of 173 at 25 °C.

#### 2.2.2. Transmission Electron Microscopy (TEM)

Transmission Electron Microscopy images were collected using a JEOL–1010 (JEOL Ltd., Tokyo, Japan), equipped with a side-mounted Gatan Orius CCD digital camera. A drop of the nanoparticles suspension was placed on a 400-mesh formvar-coated carbon grid (Agar Scientific, Stansted, UK), followed by staining with 2% phosphotungstate acid.

#### 2.2.3. Encapsulation Efficiency

The encapsulation efficiency was determined as described in Reference [32]. Free nadifloxacin concentrations were measured in the recovered medium after particle centrifugation using a Sigma 3–16 centrifuge (Sigma, Osteorode am Harz, Germany) at 5000 rpm for 15 min. Amicon Ultra-4 centrifugal filter units with ultracel-3 membrane MW cut-off of 50 kDa were used. The nadifloxacin concentration collected after centrifugation (non-encapsulated nadifloxacin) was measured using UV spectroscopy at 290 nm. The nadifloxacin encapsulation efficiency (EE) (%) was given by the difference between the total amount of nadifloxacin added for the nanoparticles preparation, and the nadifloxacin collected in solution after centrifugation to the total amount of nadifloxacin added.

### 2.3. Bacterial Strains and Growth Conditions

*S. aureus* strain EMRSA-15 was obtained from Dr. Sean Nair, University College, London. Bacteria were grown in Mueller Hinton Broth (MHB) (Sigma-Aldrich, Dorset, UK) followed by incubation at 250 rpm (for liquid cultures), at 37 °C for 18 h.

### 2.4. Eukaryotic Cell Lines and Growth Conditions

HaCaT cells were obtained from Dr. Amir Sharili, Queen Mary University, London and maintained in DMEM with 10% fetal bovine serum (FBS) (Sigma-Aldrich, Dorset, UK), supplemented with 5% penicillin-streptomycin (Sigma-Aldrich, Dorset, UK). Cells were maintained at 37 °C in 5% carbon dioxide.

### 2.5. Intracellular Infection of Keratinocytes by MRSA

Briefly, keratinocytes were seeded at 1.2 × 10^5^ cells/well in a 12-well plate and cultured overnight in DMEM with 10% FBS, without antibiotic. In parallel, MRSA strain EMRSA-15 was cultured overnight in MHB at 37 °C in an incubator shaker. One mL of overnight bacterial culture was centrifuged at 8000 rpm for three minutes and the pellet was resuspended in phosphate buffered saline (PBS) (Sigma-Aldrich, Dorset, UK). These steps were repeated three times to remove the bacterial toxin residues. Bacteria were diluted to a final concentration of approximately 10^7^ CFU/mL in DMEM with 10% FBS, without antibiotic. Aliquots of bacteria (1 mL, 10^7^ CFU/mL) were added to keratinocyte cultures after the original medium was removed. Bacteria were co-incubated with keratinocytes for three hours. Two hundred mg/L of gentamicin diluted in the medium was added and incubated for three hours. Medium containing bacteria and gentamicin were removed and cells were rinsed with PBS. A rinse step was performed because this medium contained very high concentrations of gentamicin, and therefore did not result in colony-forming units of bacteria when plated directly. Therefore, aliquots of PBS from the rinsing process were plated on nutrient agar to determine the remaining number of extracellular bacteria. Next, one mL of 0.5% Triton X-100 prepared in PBS was added to each well to lyse cells. Lysed cells were serially diluted in PBS and plated on nutrient agar (Sigma-Aldrich, Dorset, UK) for enumeration of intracellular bacteria. Uninfected cells were also subjected to the lysis procedure to confirm sterility. 

### 2.6. Antimicrobial Activities of PHMB/Nadifloxacin Nanoparticles against Intracellular MRSA

Keratinocytes were infected with MRSA using gentamicin protection assay as described in Section 2.5. Following gentamicin exposure to kill extracellular bacteria, infected cells were treated with PHMB and nadifloxacin alone, a combination of PHMB and nadifloxacin added individually to the wells, and PHMB/nadifloxacin pre-formulated as nanoparticles for three hours. Cells were then lysed with 0.5% Triton X-100. Lysed cells were serially diluted in PBS and plated on nutrient agar (Sigma-Aldrich, Dorset, UK) for enumeration of intracellular bacteria. Uninfected cells were also subjected to the lysis procedure to confirm sterility.

### 2.7. Assessment Re-Growth of MRSA

Keratinocytes were infected with MRSA, treated with gentamicin, followed by treatment with different antimicrobial formulations: PHMB and nadifloxacin alone or in combination as described above, for 24 h for over 72 h of the experiment. For every 24 h, old medium containing antimicrobials was removed and plated for colony counting and replaced with fresh medium containing antimicrobials.

### 2.8. Assessment Recovery of Infected Keratinocytes

After 72 h, cells were imaged by a DM4000B (Leica Biosystem, Wetzlar, Germany) upright microscope with the 20× objective lens. The morphology of keratinocytes was observed to evaluate recovery following infection and treatment. Also, to estimate cell viability, cells were trypsinized and counted using a hemacytometer.

### 2.9. Assessment of Nanoparticle Toxicity towards Keratinocytes

#### 2.9.1. LDH Cytotoxicity Assay

Keratinocytes were seeded at 1.2 × 10^5^ cells/well in a 12-well plate and cultured overnight in DMEM with 10% FBS, without antibiotic. Keratinocytes were exposed to increasing concentrations of PHMB and nadifloxacin alone and in combinations, for 24 h. After 24 h, 100 µL of medium were taken for measurement of LDH released by the cells using Pierce LDH™ Cytotoxicity Assay Kit (Thermo-Scientific, Rugby, UK). LDH assay was performed as described by the manufacturer. 

#### 2.9.2. Resazurin Cell Viability Assay

A resazurin assay was performed following LDH toxicity assay. The resazurin sodium salt (Sigma-Aldrich, Dorset, UK) was prepared as a stock solution at 440 μM in PBS and added to each well at 44 µM final concentrations. Plates were incubated for an additional 24 h. Optical density (OD) was then measured using a Tecan Infinite plate reader (Tecan Group Ltd., Mannedorf, Switzerland) at 550 nm and 630 nm. The OD value change (or % dye reduction) proportional to the viable cell number was used to plot the graph.

### 2.10. Statistical Analysis

All experiments were performed in triplicate. Statistical analysis was performed using one-way analysis of variance (ANOVA) followed by Tukey tests using the statistical packages Prism 6, Version 6.0 (GraphPad Prism 6.0, San Diego, CA, USA). Data is presented as means ± standard deviation (SD). Differences were considered to be statistically significant where *p* ≤ 0.05. For histogram and graphs, error bars represent standard deviations. * (*p* ≤ 0.05), *** (*p* ≤ 0.001), **** (*p* ≤ 0.0001), ns (not significant).

## 3. Results

### 3.1. Physical Characterization of PHMB/Nadifloxacin Nanoparticles

**Figure 2:**
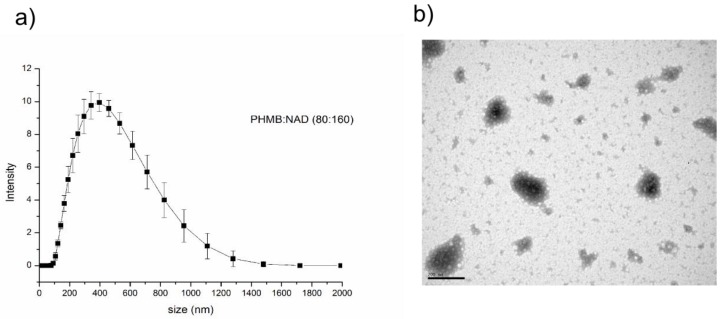
Physical characterization of the PHMB/nadifloxacin nanoparticles. (**a**) Size distribution profile obtained by dynamic light scattering analysis of PHMB/nadifloxacin nanoparticles. (**b**) Transmission electron microscopy (TEM) image of PHMB/nadifloxacin nanoparticles. The scale bar in the TEM image is 200 nm.

To prepare the PHMB/nadifloxacin nanoparticles, both compounds were mixed in to a ratio of PHMB to nadifloxacin 1:2 (*w*/*w*). The ratio was determined based on the minimum inhibitory concentrations (MIC) of the individual compound against EMRSA-15, where the MIC of PHMB and nadifloxacin were 1 mg/L and 2 mg/L, respectively [33]. Formulation of PHMB and nadifloxacin produced nanoparticles with Z-average of 291.3 ± 89.6 nm (Figure 2a), with a polydispersity index (PDI) of 0.35 ± 0.04. The zeta potential of the nanoparticles was found to be positive (+20.2 ± 4.83 mV), as expected, given that PHMB is a cationic polymer. The nanoparticles at this size have an irregular sphere shape as shown in Figure 2b. To measure the encapsulation efficiency, the free nadifloxacin was recovered in the medium following particle centrifugation and subjected to UV spectroscopy at 290 nm. The nadifloxacin encapsulation efficiency in the nanoparticle was determined to be approximately 58%.

### 3.2. Intracellular Infections of Keratinocytes by MRSA

**Figure 3:**
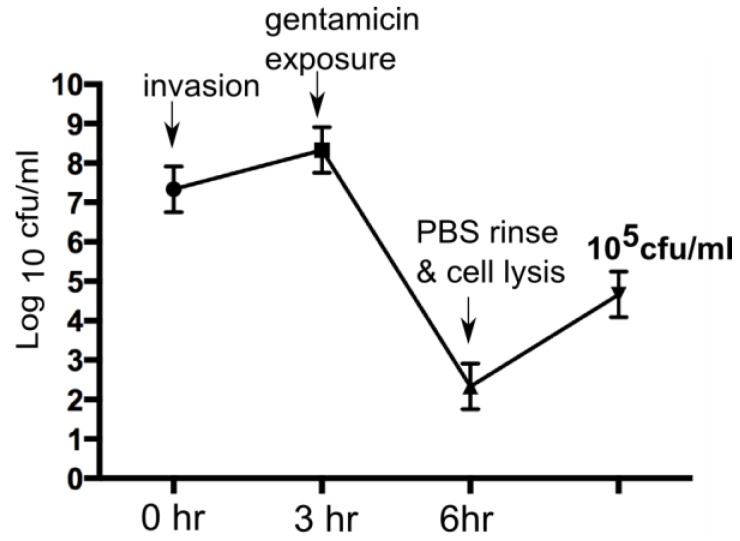
Methicillin-resistant *Staphylococcus aureus* (MRSA) invasion of keratinocytes.

We recently demonstrated that PHMB and nadifloxacin alone could kill intracellular MRSA in keratinocytes [31]. To test whether or not these nanoparticles can retain or improve the antimicrobial effects of the constituents towards intracellular MRSA, the potencies of intracellular antimicrobial effects was determined using a gentamicin protection assay as described [31]. Briefly, keratinocytes were infected with MRSA and exposed to gentamicin to kill extracellular bacteria. The MRSA strain EMRSA-15 showed consistent invasive activities towards keratinocytes, as indicated by its ability to evade gentamicin treatment. Lysis of keratinocytes following gentamicin exposure released approximately 10^5^ CFU/mL of EMRSA-15, reflecting the bacteria that were inside the keratinocytes (Figure 3).

Colony forming units (CFU) of MRSA following gentamicin exposure. After gentamicin exposure, lysis of keratinocytes released approximately 10^5^ CFU/mL of EMRSA-15.

### 3.3. Antimicrobial Activities of PHMB/Nadifloxacin Nanoparticles against Intracellular MRSA

**Figure 4:**
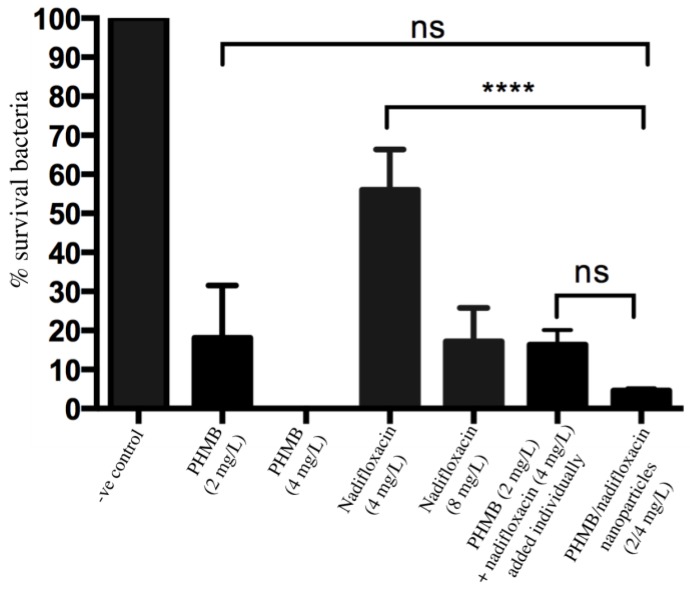
Antimicrobial activities of nanoparticles against intracellular MRSA.

To test antimicrobial activities of PHMB/nadifloxacin nanoparticles against intracellular EMRSA-15 (MRSA), both antimicrobials were tested at two and four times their MIC. Keratinocytes were infected with MRSA, as previously described. Following gentamicin exposure sufficient to kill extracellular bacteria, keratinocytes were treated with the following formulations: PHMB alone (at 2 and 4 mg/L); nadifloxacin alone (at 4 and 8 mg/L); combination of PHMB (2 mg/L) and nadifloxacin (4 mg/L), which were added individually into the wells without pre-formulation as nanoparticles; and PHMB/nadifloxacin (2:4 mg/L) pre-formulated as nanoparticles. The treatment was performed for three hours. Figure 4 shows the percentages of surviving intracellular MRSA after treatment with the antimicrobial formulations relative to the untreated infected cells. PHMB alone at 2 mg/L and 4 mg/L killed 82% and 99% of intracellular MRSA, respectively. Nadifloxacin alone at 4 and 8 mg/L killed 52% and 70% of intracellular MRSA, respectively. Combination of PHMB (2 mg/L) and nadifloxacin (4 mg/L) (added individually into the wells) killed 83% of intracellular MRSA. Finally, PHMB/nadifloxacin nanoparticles (2:4 mg/L) killed 95% of intracellular MRSA, and appeared to be more effective than the same concentrations of polymer and drug used alone, or added separately to the same culture (Figure 4); however, it is important to note that the difference was not significant.

Keratinocytes infected with MRSA were either treated with PHMB or nadifloxacin alone, or in combinations. Combinations of PHMB and nadifloxacin were added individually or pre-formulated as nanoparticles. Untreated cultures were used to establish the CFU values corresponding to 100% survival. Negative controls were the non-treated infected cells. Error bars represent standard deviations. **** (*p* ≤ 0.0001), ns (not significant).

### 3.4. Inhibition of MRSA Re-Growth

**Figure 5:**
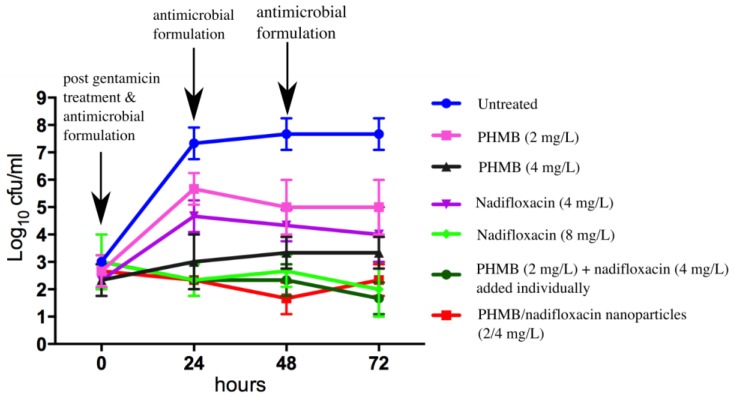
Effects of the different antimicrobials on MRSA re-growth.

To evaluate whether any of the formulations tested in this study were able to prevent re-growth of the bacteria, keratinocytes were infected with MRSA, incubated with gentamicin to kill extracellular bacteria, and treated with antimicrobial formulations as described above for 24 h. At 0, 24, 48, and 72 h, the medium containing antimicrobials formulations was sampled, plated for colony counting, and replaced with fresh medium containing antimicrobials. Re-growth of MRSA was observed for infected keratinocytes treated with PHMB alone at 2 mg/L, but not at 4 mg/L. Re-growth of MRSA was also observed for treatment with nadifloxacin alone at 4 mg/L but not at 8 mg/L. Interestingly, combinations of PHMB (2 mg/L) and nadifloxacin (4 mg/L), either added individually or pre-formulated as nanoparticles, prevented re-growth of MRSA. Therefore, a combination of PHMB and nadifloxacin at a lower dosage, either added separately as free components or pre-formulated as nanoparticles, were able to kill intracellular MRSA and prevent bacterial re-growth. Figure 5 summarizes the bactericidal activities of antimicrobials formulations following 72 h of the experiment. 

Keratinocytes infected with MRSA were either treated with PHMB or nadifloxacin alone or in combination. Combinations of PHMB and nadifloxacin were added individually or pre-formulated as nanoparticles. Untreated cultures were used as a negative control. Every 24 h for 72 h, the medium containing antimicrobials formulations was sampled, plated for colony counting, and replaced with fresh medium containing antimicrobials.

### 3.5. Recovery of Infected Keratinocytes

**Figure 6:**
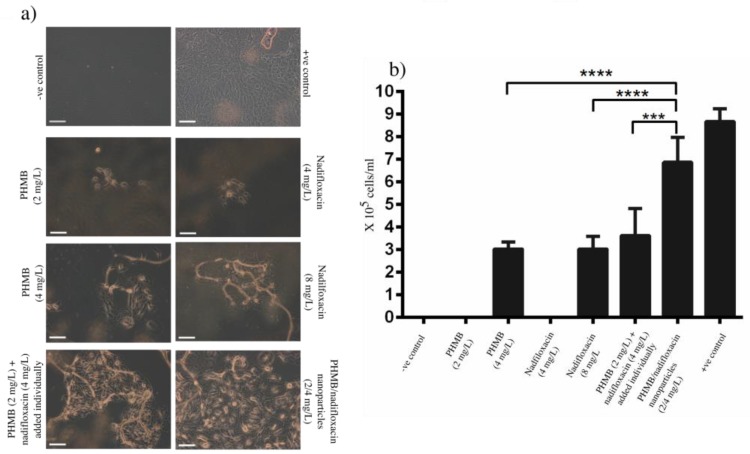
Effects of the antimicrobials on the keratinocyte’s recovery. Following antimicrobial treatment for 72 h, infected cells were: (**a**) visualized by microscopy; and (**b**) trypsinized and counted using a hemocytometer. The non-infected cells represent the positive control. The scale bar in the image is 100 µm. Bacteria added to the wells without pre-seeded host cells represent the negative control. Non-treated cells represent positive control. Error bars represent standard deviations. *** (*p* ≤ 0.001), **** (*p* ≤ 0.0001).

Invasion by virulent pathogens such as MRSA can induce pyroptosis (i.e., cell death) of host cells [34]. Therefore, an antimicrobial must not only effectively kill the bacteria, but at the same time it must help host cells recovery, or at least not inhibit this process. During the experiments performed above to investigate the prevention of the re-growth of the bacteria, we monitored the morphology of keratinocytes each day throughout the experiment. After 72 h of the treatment with different antimicrobial formulations, the recovery of infected keratinocytes was in the following order of rank: PHMB/nadifloxacin nanoparticles (2:4 mg/L) > PHMB (2 mg/L) and nadifloxacin (4 mg/L) added individually into the wells > PHMB (4 mg/L) > nadifloxacin (8 mg/L). It is surprising that cells treated with the nanoparticles showed better recovery in comparison to the cells treated with combinations of PHMB and nadifloxacin added individually into the wells, though both formulations displayed almost equal antimicrobial efficacy. Therefore, PHMB/nadifloxacin nanoparticles not only demonstrate excellent antimicrobial activities against MRSA, but also help improve recovery of infected keratinocytes. Figure 6 shows the morphology and the number of recovered keratinocytes following treatment with different antimicrobial formulations for 72 h.

### 3.6. Toxicity of PHMB/Nadifloxacin Nanoparticles

To investigate the toxicity of the formulations in relation to the free components, lactate dehydrogenase (LDH) and resazurin assays were performed. Lactate dehydrogenase is a cytoplasmic enzyme, thus, if this enzyme is released into the medium, this would indicate a disruption of the membrane integrity, and therefore a sign of the toxicity effect of the tested compound towards the host cells. In the resazurin assay, metabolically active mitochondria in viable cells reduce resazurin (purple color) into resofurin (pink color). Keratinocytes were exposed to increasing concentrations of PHMB alone (2–64 mg/L), nadifloxacin alone (4–128 mg/L) or in combinations. Combinations of PHMB and nadifloxacin were either added individually or formulated into nanoparticles. For combinations of PHMB and nadifloxacin added separately, the free components were added simultaneously in the well, whereas the nanoparticles were pre-prepared at designated concentrations and added into the cell cultures.

**Figure 7:**
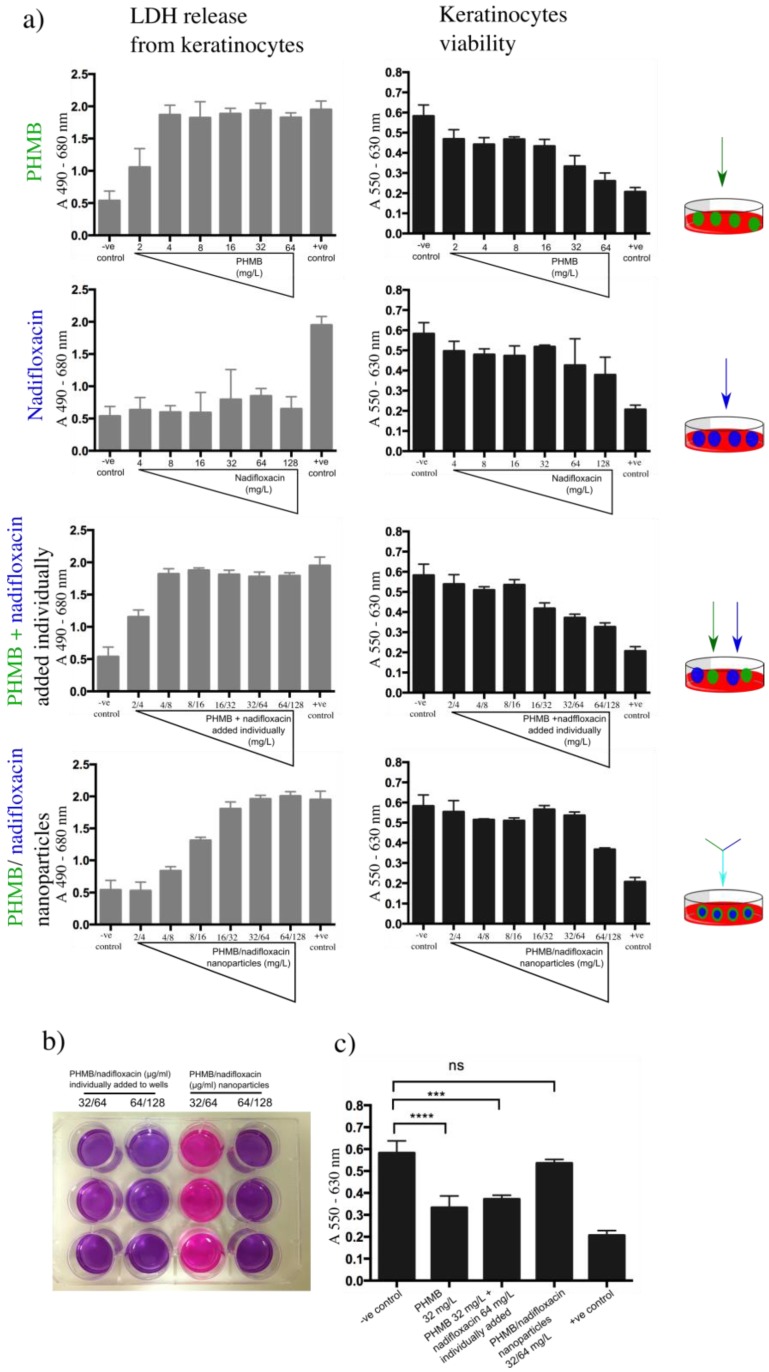
Toxicity effects of nanoparticles towards keratinocytes. Keratinocytes were exposed to increasing concentration of PHMB or nadifloxacin, alone and in combinations. The combinations were either added individually to the wells or formulated as nanoparticles. (**a**) The toxicity assessment was based on the amount of LDH released by keratinocytes, followed by evaluation of cell viability using resazurin assay. Untreated cells were used as the base for LDH released by the cells and for viability resazurin assay (negative control). Cells treated with 0.5% Triton-X 100 were used as the positive control. (**b**) Image taken on cells exposed to different antimicrobial formulation and subjected to resazurin assay. The color of resazurin added into the cells treated with 32 mg/L of PHMB and 64 mg/L nadifloxacin added individually into the wells (column 1) remained purple indicates cell death. As expected, the same was observed with higher concentrations of PHMB (64 mg/mL) and nadifloxacin (128 mg/mL) (column 2). The same effects were observed when cells were treated with PHMB alone at 32 mg/mL (image is not shown). (**c**) Statistical analysis on the viability of the cells when exposed to 32 mg/L of PHMB alone, individually added with nadifloxacin or pre-formulated as nanoparticles. No significant difference in cell viability when treated with PHMB/nadifloxacin nanoparticles at 32 mg/L compared to the non-treated cells. Error bars represent standard deviations. *** (*p* ≤ 0.001), **** (*p* ≤ 0.0001), ns (not significant).

Cells exposed to increasing concentrations of PHMB alone or PHMB added individually with nadifloxacin into the wells showed sharp increases in LDH release and a decrease in cells viability (Figure 7a). Using a resazurin assay, we observed that cell viability was affected when exposed to 32 mg/L of free PHMB, or added individually with nadifloxacin (Figure 7b). In contrast, the exposure to nadifloxacin did not induce LDH release, and the cells’ viability was not affected throughout the experiment. These findings confirm a direct relationship between the concentration of free PHMB and the toxic effect on keratinocytes. On the other hand, these results also indicate that PHMB and nadifloxacin added individually into the wells do not assemble into nanoparticles, and therefore remain as free compounds. In contrast, cells exposed to increasing concentrations of PHMB/nadifloxacin nanoparticles showed a slower release of LDH, with cell viability affected at only the highest level tested (64/128 mg/L) (Figure 7b). These results suggest that formulating PHMB and nadifloxacin as nanoparticles can reduce PHMB toxicity towards keratinocytes. The toxic effects of nanoparticles at higher concentrations could be due to the free PHMB. Further optimization to increase encapsulation efficiency can lower the amount of free PHMB in the formulation, and hence further decrease its toxic effects towards keratinocytes.

## 4. Discussion

To our knowledge, this is the first work that reports PHMB interactions with small molecule antibiotics. We hypothesized that the spontaneous formation of PHMB/nadifloxacin nanoparticles were due to direct interactions between both compounds, or through indirect interactions promoted by the environment of the compounds. These factors may contribute to the spontaneous formation of PHMB/nadifloxacin nanoparticles. PHMB is an amphiphilic polyelectrolytes compound, built of repeated hydrophobic hexamethylene groups separated by a hydrophilic biguanide segment, that can form hairpin or micelles like structures. Such structures could provide encapsulation space in its hydrophobic core for a hydrophobic compound such as nadifloxacin [35]. Since the formulation was prepared at pH 12 (nadifloxacin was dissolved in 0.1 M NaOH), there is a possibility for ionic interactions between the positively charged biguanide of the PHMB and the negatively charged carboxylate anion of nadifloxacin. This carboxyl group would be in its basic deprotonated form, COO^−^ because the pH is much higher than the pKa values of carboxylic acids which are around 4–5. In this case, we believe that the pKa of the carboxyl group would be lowered due to the stabilizing effect of the conjugated double bonds on the negative charge of the oxygen, and so at pH 12 virtually all nadifloxacin would be in its carboxylate anion form, which could promote interactions. Additionally, hydrophobic interactions could occur between the hexamethylene moiety of PHMB and the hydrophobic ring structure of nadifloxacin. Formulation at pH 12 ensured the neutrality of the tertiary nitrogens between the cycles, as the pKa values of conjugate acids of tertiary amines was lower than 12. Moreover, the lone pairs of these nitrogens are delocalized in the aromatic ring, rendering protonation of their aliphatic counterparts. Therefore, the basic neutral form of these amine nitrogens prevented any potential electrostatic repulsion of the positively charged biguanide that could destabilize the complex. Finally, there was also the possibility of formation of hydrogen bonding between the lone pairs of the oxygens and fluorine on nadifloxacin with the hydrogens covalently bound to nitrogen in the biguanide groups. Overall, these favorable interactions may explain why we achieved good encapsulation efficiency (~58%) of nadifloxacin within nanoparticles. In our previous study, we demonstrated interactions between PHMB and other molecules. For example, PHMB interactions with CpG ODN forming nanoparticles improved CpG ODN delivery into macrophages [29]. The ability of PHMB to establish interactions with drugs and oligonucleotides suggests that this polymer is a flexible platform for combinations drug reformulation.

The self-assembled PHMB/nadifloxacin nanoparticles displayed desirable physical properties that could promote uptake into mammalian cells. A monodisperse population of nanoparticles of size <500 nm is considered suitable for endocytic uptake and vesicle entrapment by the mammalian cells [36]. Additionally, the positive charge of the PHMB/nadifloxacin nanoparticles could promote electrostatic interactions with the negative charge of the cell membrane, further enhancing nanoparticles attachment on the cells’ surface and help the invagination process [37]. Furthermore, the asymmetrical spherical shape of the nanoparticles could provide large surface area for attachment onto the cells; increasing the rates of uptake into the cells [36] demonstrated that the rates of uptake for asymmetrical nanoparticles into HeLa cells are four times faster compared to the symmetrical nanoparticles.

The potent antimicrobial effects of PHMB:nadifloxacin nanoparticles against intracellular bacteria and their ability to prevent bacterial re-growth could be due to antimicrobial activity of both components and features of the particles. PHMB’s antimicrobial activities appear to be due, in part, to the membrane destabilization and cellular leakages due to the interaction of the biguanide groups with cytoplasmic membranes, lipopolysaccharides, and peptidoglycan of the bacterial cell wall [38]. Also, the hexamethylene segment can interact with phospholipids on the membrane, causing a phase separation that disturbs random distribution of lipids, further destabilizing the membrane structure [39]. Furthermore, recent findings in our laboratory demonstrated that PHMB can enter bacteria cells and condense bacterial chromosomes [28]. PHMB has a strong affinity towards DNA which is believed due to the strong electrostatic interaction between the negatively charged phosphate backbone of DNA and the cationic charged of polymer PHMB [29]. Additionally, nadifloxacin antibacterial activities is due to the inhibition of DNA gyrase, interrupting the cleavage-religation during the DNA replication process [40,41]. Thus, combination of PHMB and nadifloxacin effectively kill and prevent re-growth of the bacteria. 

Combinations of drugs have long been applied in the pharmaceutical industry as a strategy to enhance their therapeutic effect and reduce toxicities. Here we demonstrated that combinations of PHMB and nadifloxacin formulated as nanoparticles were more potent than the free antimicrobials used alone or in combination, (i.e., without pre-formulation as nanoparticles). The nanoparticles effectively killed intracellular MRSA at the lower dose, prevented re-growth of the bacteria during 72 h of treatment, improved recovery of the infected keratinocytes, and finally, showed less toxicity towards the host cells compared to other formulations tested. While this study is at an early stage, the results indicate that the nanoparticles tested can provide additional benefits that go beyond the benefits of simple co-administration. It is interesting to note that combinations of PHMB and nadifloxacin as nanoparticles showed potent antimicrobial activities towards intracellular MRSA and at the same time reduced PHMB toxicity towards the host cells. Currently, it is unknown why the nanoparticles reduced PHMB toxicity toward the mammalian cells. However, based on our understanding of the PHMB structure, we anticipated that toxicity of PHMB toward the mammalian cells could be due to the interaction of the long chain polymer, which is built of hexamethylene and biguanide moiety with cell membranes. Formulation of PHMB with nadifloxacin produced a sphere-like nanoparticles that could reduce membrane interactions and damage. Nevertheless, additive antimicrobial effects of PHMB/nadifloxacin nanoparticles against intracellular MRSA and prevention of the bacterial re-growth suggest that formulations of nanoparticles did not reduce PHMB potent activities against intracellular MRSA. This is in agreement with a previous study that showed a combination of PHMB with phosphatidylcholine maintained its antimicrobial activities against extracellular *S. aureus* and *Pseudomonas aeruginosa* with reduced toxicity effects towards fibroblast [42].

## 5. Conclusions

In conclusion, there are four main findings in this study. Firstly, PHMB can be formulated with nadifloxacin forming nanoparticles via a self-assembly method. Secondly, PHMB/nadifloxacin nanoparticles retained potency of PHMB and nadifloxacin when tested against intracellular *S. aureus*. Thirdly, PHMB/nadifloxacin nanoparticles can improve recovery of infected cells. Finally, formulations of nanoparticles can reduce the toxicity of PHMB, compared to its use either as a free component alone or in combination with nadifloxacin. Therefore, these findings encourage further investigations of PHMB/antibiotic combinations as nanoparticles formulations with improved efficacy/toxicity profiles.
